# Impact of prolonged carbapenem use-focused antimicrobial stewardship on antimicrobial consumption and factors affecting acceptance of recommendations: a quasi-experimental study

**DOI:** 10.1038/s41598-023-41710-4

**Published:** 2023-09-04

**Authors:** Jin Sae Yoo, Jeong Yong Park, Ha-Jin Chun, Young Rong Kim, Eun Jin Kim, Young Hwa Choi, Kyoung Hwa Ha, Jung Yeon Heo

**Affiliations:** 1 Department of Infectious Diseases, Ajou University School of Medicine, Worldcup-ro, 164, Yeongtong-gu, Suwon, Gyeonggi-do 16499 Republic of Korea; 2 Department of Acute Care Medicine, Ajou University School of Medicine, Suwon, Republic of Korea; 3 Department of Pharmaceutical Service, Ajou University Hospital, Suwon, Republic of Korea; 4 Department of Endocrinology and Metabolism, Ajou University School of Medicine, Ajou University School of Medicine, Worldcup-ro, 164, Yeongtong-gu, Suwon, Gyeonggi-do 16499 Republic of Korea

**Keywords:** Bacterial infection, Outcomes research

## Abstract

This study aimed to assess the impact of a prolonged carbapenem use-focused antimicrobial stewardship program (ASP) on antimicrobial consumption and clinical outcomes and to analyze factors affecting adherence to interventions. Patients prescribed carbapenems for ≥ 2 weeks received intervention. Interrupted time-series analysis was performed to compare antimicrobial consumption before and after intervention. Factors associated with non-adherence to intervention were investigated. Of 273 patients who were eligible for intervention, discontinuation or de-escalation was recommended in 256 (94.1%) and intervention was accepted in 136 (53.1%) patients. Before intervention, carbapenem consumption significantly increased to 1.14 days of therapy (DOT)/1000 patient days (PD)/month (*P* = 0.018). However, it significantly declined by − 2.01 DOT/1000 PD/month without an increase in other antibiotic consumption (*P* < 0.001). Factors affecting non-adherence to intervention were younger age (odds ratio [OR] = 0.98; 95% confidence interval [CI] 0.96–1.00), solid organ malignancy (OR = 2.53, 95% CI 1.16–5.50), and pneumonia (OR = 2.59, 95% CI 1.08–6.17). However, ASP intervention was not associated with clinical outcomes such as length of hospital stay or mortality. Prolonged carbapenem prescription-focused ASP significantly reduced carbapenem consumption without adverse outcomes. Non-adherence to interventions was attributed more to prescriber-related factors, such as attitude, than patient-related factors including clinical severity.

## Introduction

Antimicrobial resistance is becoming a threat to public health worldwide. Although it is well known that antimicrobial stewardship programs (ASPs) play a vital role in promoting the optimal use of antimicrobial agents and reducing antimicrobial resistance^1^, a stewardship program has not yet been widely introduced in clinical practice, particularly in intensive care units (ICU), long-term care facilities, and even tertiary hospitals. Many factors make it challenging to conduct an ASP in the real world, from the lack of human and material resources such as information technology, data analysis, or multidisciplinary teams to organizational culture in healthcare facilities such as communication, relational dynamics, or conflict management^2–4^. It is occasionally one of the most significant barriers to an infectious disease (ID) specialist, a medical microbiologist, or a hospital pharmacist devoting time to antimicrobial stewardship. ASP activities are often conducted by ID specialists and pharmacists, who already have a heavy workload. To overcome these barriers and efficiently use limited resources, it would be helpful to know if the ASP strategy focusing on certain antibiotics could be effective in reducing antibiotic consumption and resistance. Meanwhile, there is a concern that ID specialist recommendations such as antibiotic de-escalation or discontinuation might negatively affect critically ill patients, especially in ICU or prolonged hospital stays. This concern is based on the fact that inappropriate antibiotic therapy can be associated with a considerable increase in mortality or adverse outcomes^5^. However, prolonged prescription of broad-spectrum antibiotics could result in *Clostridium difficile* infection or adverse drug events and facilitate the spread of multidrug-resistant organisms^6,7^. Antimicrobial resistance to carbapenem among gram-negative bacteria is a major concern because it results in high attributable mortality and hospital costs while presenting few therapeutic options^8,9^. Given several issues, such as lack of human resources, patient safety, or physician acceptance for the implementation of successive ASP, reducing prolonged carbapenem prescription could be an effective way to promote the optimal use of antimicrobial agents, minimizing concerns about patient safety. Although some studies have shown that carbapenem-focused ASP intervention could reduce antimicrobial consumption and resistance without unintended clinical outcomes^10–12^, few studies have investigated the effectiveness of ASP concentrated on prolonged carbapenem prescriptions^13^. Moreover, factors that affect the acceptance of ASP recommendations, such as antibiotic de-escalation or discontinuation, have not been fully elucidated^14,15^ and may differ among institutions. Therefore, this study aimed to assess the impact of prolonged carbapenem use-focused ASP on changes in carbapenem and other antimicrobial consumption in addition to clinical outcomes and to analyze factors affecting physicians’ adherence to ASP interventions.

## Methods

### Study design and period

A quasi-experimental study was performed between September 1, 2019, and August 31, 2021, consisting of 12 months each of the pre- and post-intervention periods. The month immediately after ASP initiation was considered the intervention period. Individuals in the pre-intervention period consisted of patients who were prescribed carbapenem antibiotics, including ertapenem, imipenem-cilastatin, and meropenem, for ≥ 14 days. Individuals in the post-intervention period consisted of patients who received ASP feedback for carbapenem prescriptions for ≥ 14 days. This study was based on a large single-hospital experience at a 1200-bed university-affiliated teaching hospital, and was conducted following approval from the Human Research Protection Center (HRPC), the Institutional Ethics Committee of Ajou University hospital. The study was performed in accordance with relevant guideline, and informed consent was waived after the HRPC committee had reviewed the study protocol. Because this retrospective study had a minimal risk and the analysis used anonymous data.

### Study setting and intervention

Before this study, the study hospital had an antibiotic restriction policy that required electronic approval from ID experts to continue a prescription of restricted antibiotic agents within 5 days of initiation, a concept similar to a preauthorization. Antibiotic prescription approval is required for fourth-generation cephalosporins, anti-pseudomonal penicillins, carbapenems, and glycopeptide antibiotics. After approval, antibiotics could be prescribed without further intervention for up to 28 days. From September 1, 2020, a prospective audit and feedback (PAF)-based ASP targeting prolonged carbapenem prescriptions was provided across the entire hospital by an ASP team consisting of one ID specialist and two clinical pharmacists. A prolonged carbapenem prescription was defined as a case where one had been consecutively receiving a carbapenem prescription for at least 14 days. Physicians prescribing carbapenem antibiotics to a patient for 14 days ordered an automatic, compulsory consultation with the ASP team through an electronic medical record system. Upon consultation, a clinical pharmacist recorded the patient’s diagnoses, antibiotic prescription history, microbiological data, and laboratory studies. He also evaluated the appropriateness of the carbapenem prescription based on predefined criteria for the patient’s clinical status and microbiological findings (Supplementary Fig. 1). The pharmacist provided an initial assessment of the appropriateness of maintaining the carbapenem prescription, which was then followed by feedback from an ID specialist regarding how to adjust the current antibiotic therapy, including de-escalation or discontinuation. Adherence, defined in this study as adhering to feedback from the ASP within 3 days of consultation, was not obligated. Patients diagnosed with hematologic malignancies or those with a history of solid organ transplant or autologous/allogeneic stem cell transplant were excluded from the first 3 months of intervention.

### Data collection and outcomes

The collected data consisted of four categories: (1) patient demographic data, such as age, sex, patient location on consultation date (general ward or ICU), and admitting department (medical or surgical); (2) clinical-microbiological data, such as the major diagnosis leading to admission, clinical severity, and suspected infectious focus at the time of carbapenem prescription, and microbiology results during the patient’s clinical course; clinical severity was classified into a severity grade depending on admission to the ICU, use of mechanical ventilation, or administration of inotropes, with severity grade elevating by 1 with concomitant corresponding factors; (3) clinical outcome data, including length of hospital stay and in-hospital mortality; and (4) antimicrobial consumption of four different antibiotic classes (third-generation cephalosporin, fourth-generation cephalosporin, piperacillin-tazobactam, and carbapenem) across the entire hospital, calculated as days of therapy (DOT)/1000 patient-days (PD). With the exception of ceftolozane-tazobactam, fifth generation cephalosporins such as ceftaroline or antibiotics with novel beta-lactamase inhibitors (such as avibactam or relebactam) were not available in Korea in the time of the study period and were not included in the analysis. The consumption of ceftolozane-tazobactam was extremely small due to high cost and therefore was not included in the analysis as well. Response to intervention was classified as either adherence or non-adherence, defined as de-escalation or discontinuation of antibiotic therapy within 3 days of feedback.

### Statistical analysis

Descriptive and comparative studies of the data from the pre- and post-intervention periods were performed. Quantitative data are reported as mean ± standard deviation (SD), and categorical data as frequencies (%). Student’s t-test and the chi-square test, or Fisher’s exact test, were used for comparing quantitative and categorical data, respectively. To understand the differences between the intervention-adherence and non-adherence groups in a subgroup analysis of the post-intervention period, a logistic regression analysis was performed to investigate the factors affecting non-adherence to the antimicrobial stewardship intervention. Variables associated with intervention adherence in the univariate analysis (*P* < 0.10) or that were likely to be clinically relevant were included in the final model. Interrupted time-series regression analysis using autoregressive integrated moving average models was performed to measure the effect of ASP on the antimicrobial consumption trend^16^. Changes in level (difference between the observed outcome at the first point of intervention and the outcome predicted by the pre-intervention trend) and trend (difference between pre- and post-intervention slopes) were estimated. We also included step and ramp intervention function to test for both an immediate and sustained change^17^. Statistical analyses were performed using the SPSS software (version 25; IBM, New York, the United State [U.S.]) and Statistical Analysis Software (version 9.4; SAS Institute, Inc, Cary, the U.S.).

## Results

### Baseline characteristics of the study patients

**Figure 1:**
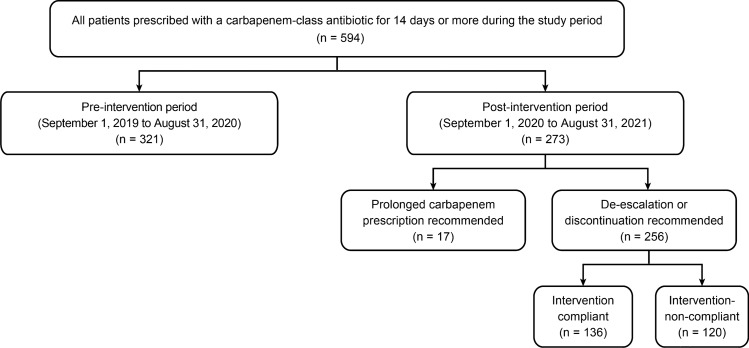
Study flow for a quasi-experimental and univariate/multivariate analysis.

A total of 594 patients who were prescribed carbapenem for ≥ 14 days during the study period were included in the analysis. A PAF-based ASP was provided to 273 patients receiving carbapenem consecutively for ≥ 14 days during the post-intervention period. Meanwhile, 321 patients were identified as having a prolonged carbapenem prescription for at least 14 days during the pre-intervention period (Fig. 1). The baseline features of patients in the pre- (321 patients) and post-intervention (273 patients) groups are summarized in Supplementary Table 1. Patient demographics, the distribution of admitting departments, and the patient location at the time of intervention were similar between the groups. In both groups, solid organ malignancy and pneumonia were the most and second-most frequent primary diagnoses on admission, respectively, accounting for slightly less than half of the included patients.

### Clinical and microbiological outcomes

The distribution of suspected infectious foci did not differ significantly between the pre- and post-intervention groups (Table 1). Prolonged carbapenem therapy was most frequently directed against suspected respiratory tract infections in both groups. Notably, approximately 10% of the study patients received prolonged carbapenem prescriptions despite having no suspected infectious focus. In both groups, bacteria classified under the order *Enterobacterales* were the most frequently isolated pathological organisms. However, the post-intervention group demonstrated a significantly greater proportion of isolated *Enterobacterales* susceptible to a third-generation cephalosporin (*P* = 0.004) and a significantly lower proportion of unidentified gram-negative bacilli (*P* = 0.009). The length of hospital stay (52.33 ± 43.58 days vs. 53.34 ± 37.66 days, *P* = 0.763) and mortality rate during hospitalization (28.0% vs. 26.4%, *P* = 0.650) were not significantly different between the pre- and post-intervention groups.

**Table 1:** Clinical and microbiological features in the patients on pre-intervention and post-intervention period.

	Pre-intervention	Post-intervention	*P*-value
	n = 321	n = 273	
Age (mean ± SD), years	65.95 ± 14.94	65.84 ± 14.19	0.931
Sex, n (%)			
Male	201 (62.6)	160 (58.6)	0.319
Suspected infectious foci at the time of carbapenem prescription, n (%)			
Respiratory tract	164 (51.1)	122 (44.7)	0.120
Genitourinary tract	37 (11.5)	42 (15.4)	0.168
Intraabdominal	50 (15.6)	48 (17.6)	0.512
Bone and soft tissue	19 (5.9)	13 (4.8)	0.534
Bloodstream	13 (4.0)	13 (4.8)	0.672
Central nervous system	6 (1.9)	3 (1.1)	0.444
Head and neck	3 (0.9)	3 (1.1)	0.842
Multifocal site	1 (0.3)	1 (0.4)	0.909
No focus	28 (8.7)	28 (10.3)	0.524
Microorganisms, n (%)			
*Enterobacterales*, third CEP sensitive	22 (6.9)	38 (13.9)	0.004
*Enterobacterales*, third CEP resistant	80 (24.9)	71 (26.0)	0.762
Non-fermentative GNB^a^, not MDRO	17 (5.3)	24 (8.8)	0.094
Non-fermentative GNB^a^, MDRO	30 (9.3)	22 (8.1)	0.580
Others	6 (1.9)	6 (2.2)	0.777
No GNB identified	166 (51.7)	112 (41.0)	0.009
Length of hospital stay (mean ± SD), days	52.33 ± 43.58	53.34 ± 37.66	0.763
In-hospital mortality, n (%)	90 (28.0)	72 (26.4)	0.650

*SD* standard deviation, *third CEP* third-generation cephalosporin, *GNB* gram-negative bacilli, *MDRO* multidrug-resistant organism.

^a^Includes *Pseudomonas aeruginosa* and *Acinetobacter baumannii.*

### Changes in antimicrobial consumption

**Figure 2:**
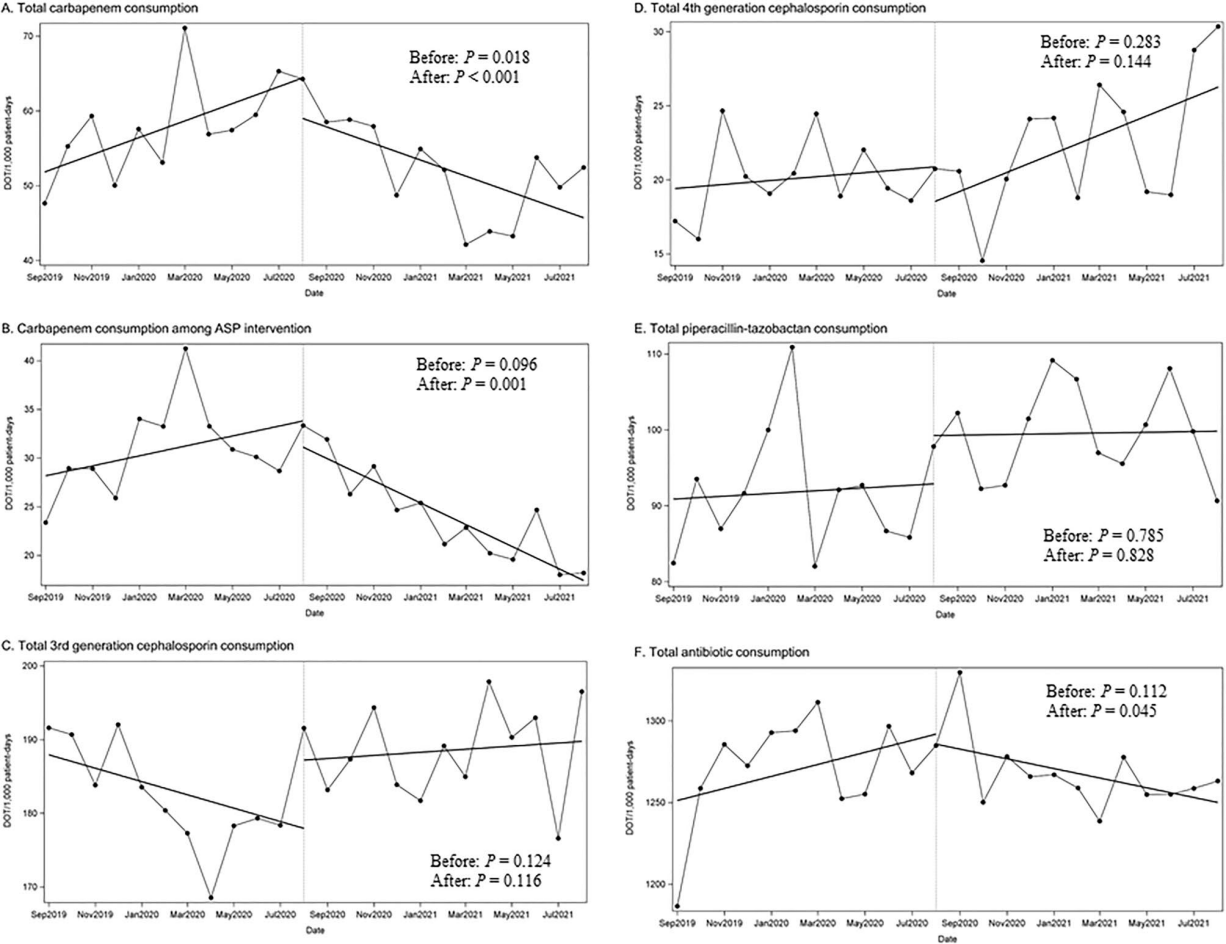
Changes in the trend of antibiotic consumption before and after antimicrobial stewardship intervention. Unit of antibiotic consumption is days of therapy (DOT)/1000 patient-day. Unit of observation is months. Dotted line represents initiation of intervention after 12 months of observation. (**A**) Total amount of carbapenem consumption through the hospital. (**B**) Amount of carbapenem consumption in patients requiring antimicrobial stewardship intervention. (**C**) Total amount of third-generation cephalosporin consumption through the hospital. (**D**) Total amount of fourth-generation cephalosporin consumption through the hospital. (**E**) Total amount of piperacillin-tazobactam consumption through the hospital. (**F**) Total antibiotic consumption throughout the hospital.

Before the implementation of the PAF-based antimicrobial stewardship intervention, the total carbapenem antibiotic consumption had a significantly increasing trend of 1.14 DOT/1,000 PD/month (*P* = 0.018) (Fig. 2 and Supplementary Table 2). However, the non-significant decreasing trend of − 7.42 DOT/1000 PD/month was immediately observed after the initiation of the intervention (*P* = 0.104), and a significantly decreasing trend of − 2.01 DOT/1000 PD/month was maintained after the intervention period (*P* < 0.001). In the subgroup of patients prescribed carbapenem antibiotics for ≥ 14 days, carbapenem consumption significantly decreased by − 1.547 DOT/1000 PD/month after ASP intervention (*P* = 0.001). The consumption of piperacillin-tazobactam, fourth-generation cephalosporin, and third-generation cephalosporin did not significantly change, but total antibiotic consumption decreased by − 6.696 DOT/1000 PD/month after ASP intervention (*P* = 0.045).

### Factors affecting intervention adherence

Among the 273 patients included in the post-intervention group, 256 (93.8%) recommended discontinuation or de-escalation of prolonged carbapenem prescriptions, and 136 (53.1%) patients adhered to the intervention. The clinical and microbiological characteristics of the intervention non-adherence group were compared with those of the adherence groups (Table 2). Patients in the non-adherence group were more likely to have solid organ malignancy (odds ratio [OR] = 1.99, 95% confidence interval [CI] 1.14–3.64) as the primary diagnosis leading to admission. Intervention adherence tended to be significantly lower in patients in medical departments than in those in surgical departments (OR = 1.99, 95% CI 1.11–3.59). Prolonged empirical therapy with carbapenem without microbiological evidence was less likely to adhere to ASP intervention (OR = 2.00, 95% CI 1.04–3.87). In contrast, genitourinary tract infection was significantly associated with higher intervention adherence than others (OR = 0.47, 95% CI 0.23–0.95). However, clinical outcomes, such as length of hospital stay and mortality, were not significantly different between the adherence and non-adherence groups. On multivariate logistic regression analysis, solid organ malignancy (OR = 2.53, 95% CI 1.16–5.50), and pneumonia (OR = 2.59, 95% CI 1.08–6.17) as primary diagnoses at admission were independent predictors of non-adherence to ASP intervention (Table 3).

**Table 2:** Comparison of clinical and microbiological characteristics between intervention-adherence and non-adherence group.

	Adherence	Non-adherence	Odds ratio (95% CI)	*P*-value
	n = 136	n = 120		
Age (mean ± SD), years	68.03 ± 12.92	64.68 ± 14.96	0.98 (0.97–1.00)	0.058
Sex				
Male, n (%)	77 (56.6)	71 (59.2)	1.11 (0.68–1.83)	0.705
Primary diagnoses at admission, n (%)				
Solid organ malignancy	29 (21.3)	42 (35.0)	1.99 (1.14–3.64)	0.016
Pneumonia	20 (14.7)	29 (24.2)	1.85 (0.98–3.48)	0.057
Chronic liver disease/cirrhosis	12 (8.8)	11 (9.2)	1.04 (0.44–2.46)	0.924
Cerebrovascular/neurologic diseases	12 (8.8)	5 (4.2)	0.45 (0.15–1.32)	0.135
Cardiovascular disease	11 (8.1)	5 (4.2)	0.49 (0.17–1.47)	0.196
Urosepsis	15 (11.0)	6 (5.0)	0.43 (0.16–1.13)	0.087
Peritonitis	10 (7.4)	6 (5.0)	0.66 (0.23–1.88)	0.440
Others	27 (19.9)	16 (13.3)	0.62 (0.32–1.22)	0.164
Departments				
Medicine, n (%)	94 (69.1)	98 (81.7)	1.99 (1.11–3.59)	0.022
ICU, n (%)	50 (36.8)	36 (30.0)	0.74 (0.44–1.24)	0.253
Infectious foci at the time of carbapenem prescription, n (%)				
Respiratory tract	57 (41.9)	59 (49.2)	1.34 (0.82–2.20)	0.245
Genitourinary tract	28 (20.6)	13 (10.8)	0.47 (0.23–0.95)	0.036
Intraabdominal	19 (14.0)	24 (20.0)	1.54 (0.80–2.98)	0.200
Others	18 (13.2)	10 (8.3)	0.60 (0.26–1.35)	0.214
No focus	14 (10.3)	14 (11.7)	1.15 (0.53–2.52)	0.726
^a^Clinical severity grade, n (%)				
0–1	89 (65.4)	89 (74.2)		
2–3	47 (34.6)	31 (25.6)	0.66 (0.38–1.13)	0.131
Type of therapy				
Empirical, n (%)	104 (76.5)	104 (86.7)	2.00 (1.04–3.87)	0.039
Microorganisms, n (%)				
*Enterobacterales*, third CEP resistant	37 (27.2)	26 (21.7)	0.74 (0.42–1.32)	0.305
Non-fermentative GNB	25 (18.4)	18 (15.0)	0.78 (0.40–1.52)	0.471
No GNB identified	50 (36.8)	59 (49.2)	1.66 (1.01–2.74)	0.046
Length of hospital stay (mean ± SD), days	54.40 ± 40.81	50.90 ± 34.31	1.00 (0.99–1.00)	0.460
In-hospital mortality, n (%)	31 (22.8)	37 (30.8)	1.51 (0.87–2.64)	0.147

^a^Severity grade grade 0 to grade 3, elevated by 1 when each criterion was met: (1) admission to ICU, (2) use of mechanical ventilation, (3) administration of inotropics.

*CI* confidence interval, *SD* standard deviation, *ICU* intensive care unit, third *CEP* third-generation cephalosporin, *GNB* gram-negative bacilli.

=

**Table 3:** Multivariate analysis of variables affecting non-adherence for antimicrobial stewardship intervention.

	Odds ratio	95% CI	*P*-value
Age	0.98	0.96–1.00	0.032
Sex			
Male	0.95	0.54–1.69	0.867
Primary diagnoses at admission			
Solid organ malignancy	2.53	1.163–5.50	0.019
Pneumonia	2.59	1.08–6.17	0.032
Urosepsis	1.21	0.29–5.03	0.796
Chronic liver disease/cirrhosis	0.88	0.28–2.71	0.817
Peritonitis	1.04	0.26–4.14	0.961
Departments			
Medicine vs. surgery	1.91	0.87–4.16	0.106
ICU	1.99	0.56–7.07	0.287
Infectious foci at the time of carbapenem prescription			
Respiratory tract	1.04	0.34–3.20	0.952
Genitourinary tract	0.75	0.21–2.72	0.659
Intraabdominal	2.34	0.72–7.57	0.157
No focus	0.87	0.23–3.25	0.831
^a^Clinical severity grade, n (%)			
2–3 versus 1–2	0.36	0.10–1.33	0.125
Type of therapy			
Empirical	1.77	0.66–4.74	0.259
Microorganisms			
*Enterobacterales*, third CEP resistant	1.63	0.56–4.76	0.375
Non-fermentative GNB	0.92	0.34–2.57	0.874
No GNB identified	1.18	0.50–2.82	0.705
Length of hospital stay	1.00	0.99–1.01	0.639
In-hospital mortality	1.47	0.76–2.84	0.254

*CI* confidence interval, *SD* standard deviation, *ICU* intensive care unit, *third CEP* third-generation cephalosporin, *GNB* gram-negative bacilli.

^a^Severity grade grade 0 to grade 3, elevated by 1 when each criterion was met: (1) admission to ICU, (2) use of mechanical ventilation, (3) administration of inotropics.

## Discussion

In this quasi-experimental study, ASP intervention focusing on prolonged carbapenem prescription significantly decreased total carbapenem consumption without increasing consumption of other classes of antibiotics targeting gram-negative bacteria. Furthermore, adherence to antimicrobial stewardship interventions had no significant effect on clinical outcomes such as length of hospital stay or mortality. The effect of ASP on reducing antimicrobial consumption has been consistently demonstrated^18,19^. However, concerns regarding the decrease in consumption of a certain antibiotic being mitigated by increased consumption of other antibiotics, called the ballooning effect, have been documented^12,20^. In this study, such an effect was not observed, which aligns with the main goal of ASP: reducing unnecessary antibiotic prescriptions. Given an average length of hospital stay of approximately 50 days, or in-hospital mortality rate of > 30%, the patients were more likely to be critically ill. In these critically ill patients, a reduction in carbapenem prescription without a ballooning effect could be achieved. This suggests that PAF-based ASP could be inevitable in controlling the prolonged use of broad-spectrum antibiotics, such as carbapenems. The study hospital already had an antibiotic restriction policy that required electronic approval from ID experts to continue broad-spectrum antibiotics within 5 days of initiation, a concept similar to preauthorization. However, the consumption of broad-spectrum antibiotics has been steadily increasing despite the introduction of restrictions to limit the use of specific antibiotics^21^. Therefore, PAF intervention was additionally performed through a multidisciplinary approach. Total antibiotic consumption also decreased following the implementation of carbapenem-focused ASP: this reduction cannot be explained by decreased carbapenem consumption alone, and whether this overall decrease in antibiotic consumption itself, rather than the effect of ASP, more heavily influenced the result of this study is unclear. Further continuous monitoring of antibiotic consumption is necessary to elucidate on this possible confounding factor.

Implementation of robust ASP that adequately covers every aspect of the program requires sufficient human and material resources, which is often not available in large parts of the world^22,23^. A study in South Korean hospitals reported that 1.20 full-time equivalents per 100 beds were required to perform extensive ASP activities on all hospitalized patients^3^, but a survey in 2018 found that only 6.0% had full-time workers for ASP^24^, with the rest of the hospitals having, on average, 2–3 infectious disease specialists available to oversee ASP on part-time basis. To implement and sustain an effective ASP in resource-limited setting, a more focused ASP tailored to each hospital’s need and capability may be feasible. In this study, the ASP focused on prolonged carbapenem prescription given to patients in critical conditions or under long hospital stays, which in most cases was not expected to provide foreseeable benefit when continued beyond a certain time period. This focused activity, although different from conventional ASP, did contribute to decline in overall carbapenem consumption while not straining the resources available to the hospital. Such approach in resource-limited settings, as demonstrated in this study, may be an effective alternative strategy to conventionally operated ASP.

Notably, prolonged carbapenem-focused ASP had no unintended consequences such as increased hospital stays or in-hospital mortality. In this study, 93.8% of the patients continuously receiving carbapenem therapy required antibiotic discontinuation or de-escalation on day 14, and half of those had no identified gram-negative bacteria. Approximately 10% of the patients were receiving prolonged carbapenem therapy without a suspected focus of infection. Therefore, prolonged carbapenem-prescription-focused ASP was prioritized in the study hospital. Previous studies showed inconsistent findings for antibiotic de-escalation in critically ill patients^25,26^. In a systematic review, uncertainty was observed regarding the effectiveness and safety of antibiotic de-escalation in patients with sepsis^27^. However, shortening the duration of antibiotic therapy through antimicrobial stewardship interventions can lead to similar clinical outcomes compared to no intervention^28,29^. Moreover, recently published data have demonstrated that the de-escalation of empirical broad-spectrum antibiotics is safe in critically ill patients with neutropenic fever receiving stem cell transplantation or culture-negative severe pneumonia^30–32^. Although only 256 patients receiving prolonged carbapenem prescriptions for at least 14 days were identified over a 1-year intervention period, we could achieve our goal of reducing the total carbapenem consumption across the hospital. This suggests that one of the most significant challenges to the optimal use of antimicrobial agents is the prolonged prescription of broad-spectrum antibiotics in critically ill patients.

An overall adherence of 53.1% to recommendations regarding the discontinuation of carbapenem therapy was observed in this study. While ASP is now recognized as an essential component for reducing unnecessary antibiotic prescriptions and improving patient outcomes in hospital settings, physician adherence to antimicrobial stewardship interventions is rarely evaluated. A study surveying 46 hospitals in the U.S. reported that 96% of the surveyed hospitals implemented ASP, but only 52% monitored adherence^33^. However, it is important to evaluate the acceptance of ASP recommendations as well as to measure antibiotic consumption and resistance in tracking, which is regarded as a core element of hospital ASP^34^. Studies that evaluated compliance with antimicrobial stewardship interventions reported non-adherence rates ranging from 5.9 to 33.3%^14,15,35,36^. PAF is labor-intensive and depends on the prescriber’s voluntary compliance. Thus, it must be fully understood which drivers are willing to accept ASP recommendations for successful implementation. In this study, the acceptance of an antimicrobial stewardship intervention was lower than that reported in previous studies. It is unfeasible to compare these acceptance rates on a head-to-head basis because of the wide variability in the method, timing of the intervention, and evaluation time after intervention initiation. However, factors affecting non-adherence to ASP recommendations indicate that prescriber-related factors, such as knowledge and attitude, appear to be considerably related to non-adherence, together with patient-related factors such as underlying conditions and clinical severity. Unless clinical improvement was observed during the study period, prescribers were hesitant to reduce or discontinue antibiotics. Therefore, the relatively low adherence rate in this study suggests several challenges in reporting, education, and communication with clinicians. Interestingly, departments of surgery showed significantly higher adherence to antimicrobial stewardship interventions than departments of medicine. In contrast to this study, a retrospective analysis of an antimicrobial stewardship intervention in Singapore reported an overall ASP adherence rate of 81.9% and found lower odds of adherence in surgical unit patients receiving carbapenems^37^. The study implied that the strict hierarchical organization of surgical teams might contribute to following team leaders’ decisions rather than accepting ASP recommendations. A similar study in Canada also found that surgical departments were less likely to accept antimicrobial stewardship interventions (85.7% for general medicine vs. 70.5% for surgery)^15^. A Canadian study noted that intervention adherence among surgical departments improved if the patient was concurrently reviewed by an ID physician. This association between concurrent consultation with an ID physician and improved compliance with ASP has been reported in another study reporting the impact of ASP on *Staphylococcus aureus* bacteremia^38^. In our study, the majority of the patients in the departments of surgery were formally consulted by an ID physician before receiving intervention regarding treatment de-escalation, which may have led to an improved adherence rate compared to the departments of medicine, where most patients were not reviewed by an ID physician beyond the initial approval of carbapenem prescription. Active involvement or consultation with ID physicians in patient care before antimicrobial stewardship intervention may lead to improved adherence and a further reduction in carbapenem consumption.

The quasi-experimental design and interrupted time-series analysis of antimicrobial consumption are a major strength of this study, as they tie causality (in this case, ASP) to observed outcomes (antibiotic consumption and clinical outcomes) more strongly than retrospective reviews, resulting in higher internal validity^39^. However, given that the regression to mean or maturation effect could be introduced as a bias in an intervention study, it may be too early to conclude that prolonged carbapenem-prescription-focused ASP has a beneficial effect on reducing antibiotic consumption without unintended consequences^40^. Thus, it is necessary to evaluate the long-term effects of prolonged carbapenem-prescription-focused ASP. Further research is warranted to address additional metrics associated with ASP tracking, such as *Clostridioides difficile* infection or antimicrobial resistance.

In conclusion, prolonged carbapenem prescription-focused ASP in a resource-limited setting significantly reduced total amount of carbapenem consumption across the hospital without adversely affecting clinical outcomes. Adherence to the interventions was not relatively high, especially among patients with solid organ cancer and pneumonia. It seems to be attributed to prescriber-related factors, such as knowledge and attitude, as well as patient-related factors, such as underlying conditions and clinical severity. Understanding prescriber-related factors and active consultations for critically ill patients who are hospitalized in medical departments are warranted to promote ASP. The effectiveness of targeted interventions, such as prolonged prescription of other broad-spectrum antibiotics or duration of therapy in patients with specific infections, on reducing antibiotic use needs to be examined further.

### Supplementary Information

Supplementary Information.

## Data Availability

The datasets used and/or analysed during the current study available from the corresponding author on reasonable request.
