# Supplementary material for: Impact of prolonged carbapenem use-focused antimicrobial stewardship on antimicrobial consumption and factors affecting acceptance of recommendations: a quasi-experimental study
Source: Sci Rep. 2023 Sep 4;13:14501. doi: 10.1038/s41598-023-41710-4 (PMC10477184; doi:10.1038/s41598-023-41710-4)
Supplement: Supplementary file 1 — Supplementary Information. [file 41598_2023_41710_MOESM1_ESM.docx]

**Impact of prolonged carbapenem use-focused antimicrobial stewardship on antimicrobial consumption and factors affecting acceptance of recommendations: A quasi-experimental study**

Jin Sae Yoo^1, 2^, Jeong Yong Park^3^, Ha-Jin Chun^3^, Young Rong Kim^1^, Eun Jin Kim^1^, Young Hwa Choi^1^, Kyoung Hwa Ha^4,*^, Jung Yeon Heo^1,*^

^1^Department of Infectious Diseases, Ajou University School of Medicine, Suwon, Republic of Korea

^2^Department of Acute Care Medicine, Ajou University School of Medicine, Suwon, Republic of Korea
^3^Department of Pharmaceutical Service, Ajou University Hospital, Suwon, Republic of Korea
^4^Department of Endocrinology and Metabolism, Ajou University School of Medicine, Suwon, Republic of Korea

**TABLE S1** Comparison of baseline characteristics between the patients in pre-intervention and post-intervention period

|  | **Pre-intervention** | **Post-intervention** | ***P*-value** |
| --- | --- | --- | --- |
|  | **n = 321** | **n = 273** |  |
| Age group, n (%) |  |  |  |
| <65 | 133 (41.4) | 117 (42.9) | 0.726 |
| 65–74 | 78 (24.3) | 65 (23.8) | 0.889 |
| ≥75 | 110 (34.3) | 91 (33.3) | 0.810 |
| Primary diagnoses at admission, n (%) |  |  |  |
| Solid organ malignancy | 87 (27.1) | 75 (27.5) | 0.920 |
| Pneumonia | 57 (17.8) | 54 (19.8) | 0.528 |
| Chronic liver disease/cirrhosis | 18 (5.6) | 24 (8.8) | 0.131 |
| Urosepsis | 23 (7.2) | 21 (7.7) | 0.807 |
| Peritonitis | 11 (3.4) | 18 (6.6) | 0.074 |
| Hematologic malignancy | 0 | 6 (2.2) | 0.008 |
| Cerebrovascular/neurologic diseases | 23 (7.2) | 16 (5.9) | 0.414 |
| Infections involving central nervous system | 4 (1.2) | 6 (2.2) | 0.369 |
| Pancreaticobiliary disease | 21 (6.5) | 8 (2.9) | 0.042 |
| Soft tissue and joint disease | 24 (7.5) | 11 (4.0) | 0.070 |
| Cardiovascular disease | 15 (4.7) | 13 (4.8) | 0.959 |
| Multiple trauma | 11 (3.4) | 4 (1.5) | 0.129 |
| Gastrointestinal bleeding and infection | 10 (3.1) | 6 (2.2) | 0.491 |
| Others | 17 (5.3) | 11 (4.0) | 0.468 |
| Departments, n (%) |  |  |  |
| Departments of medicine | 238 (74.1) | 201 (73.6) | 0.886 |
| Departments of surgery | 83 (25.9) | 72 (26.4) |  |
| Patient location at time of intervention, n (%) |  |  |  |
| General ward | 207 (64.5) | 178 (65.2) | 0.856 |
| Intensive care unit | 114 (35.5) | 95 (34.8) |  |

**TABLE S2** Changing trend of antibiotic consumption before and after implementation of prospective audit and feedback intervention

|  | **Before intervention** | | **Intervention*^a^*** | | **After intervention*^b^*** | |
| --- | --- | --- | --- | --- | --- | --- |
|  | **ß*^c^*** | ***P-*value** | **ß** | ***P-*value** | **ß** | ***P-*value** |
| Total carbapenem consumption | 1.144 | 0.018 | −7.415 | 0.104 | −2.010 | <0.001 |
| Carbapenem consumption among ASP intervention | 0.512 | 0.096 | −3.574 | 0.228 | −1.547 | 0.001 |
| Total third CEPs consumption | −0.908 | 0.124 | 7.621 | 0.185 | 1.316 | 0.116 |
| Total fourth CEPs consumption | 0.133 | 0.283 | −3.163 | 0.268 | 0.609 | 0.144 |
| Total piperacillin/tazobactam consumption | 0.177 | 0.785 | 6.961 | 0.280 | −0.198 | 0.828 |
| Total antibiotic consumption | 3.687 | 0.112 | −5.799 | 0.793 | −6.696 | 0.045 |

*^a^*Intervention means one month immediately after initiation of prospective audit and feedback intervention.

*^b^*After intervention means whole intervention period of 12 months after implementation of prospective audit and feedback intervention.

*^c^*The unit of β coefficients is days of therapy (DOT)/1,000 patient-days.

Abbreviations: ASP, antimicrobial stewardship program; third CEPs, third generation cephalosporins; fourth CEPs, fourth generation cephalosporins.





**FIG S1** Schematization of prospective audit and feedback-based antimicrobial stewardship program
